# The role of positive affect in the relationship between neuroticism, self-esteem, and emotional clarity in adolescents

**DOI:** 10.3389/fpsyg.2026.1717007

**Published:** 2026-03-24

**Authors:** Nieves-Fátima Oropesa-Ruiz, Noelia Navarro Gómez, María-Araceli Pérez-García, José Manuel Martínez-Vicente

**Affiliations:** 1 Department of Psychology, University of Almería, La Cañada de San Urbano, Almería, Spain; 2 Department of Psychology, University of Málaga, Málaga, Spain

**Keywords:** adolescence, subjective well-being, self-esteem, emotional intelligence, personality development

## Abstract

**Introduction:**

The relationship between neuroticism and emotional clarity has been a prominent topic of research in the context of adult development and well-being. However, there is a scarcity of studies examining this relationship in adolescents, especially those that consider the protective roles of self-esteem and positive affect.

**Objective:**

The primary aim of this study was to analyze how positive affect and self-esteem influence the associations between neuroticism and emotional clarity in adolescents, assessing both mediating and moderating effects.

**Method:**

A sample of 742 secondary school adolescents (aged 13–19) completed the Neuroticism subscale from the Big Five Inventory, the Emotional Clarity subscale of the Trait Meta-Mood Scale, the Rosenberg Self-Esteem Scale, and the Positive Affect Scale.

**Results:**

The analyses confirmed the four hypotheses. Self-esteem acted as a statistical mediator in the relationship between neuroticism and emotional clarity, such that higher levels of neuroticism were associated with lower self-esteem, which in turn was associated with lower emotional clarity. Positive affect moderated both the relationship between neuroticism and emotional clarity and the relationship between neuroticism and self-esteem, attenuating the strength of these negative associations. The integrated model combining the mediation and moderation 36 effects showed a good fit, supporting the proposed theoretical framework.

**Conclusion:**

These findings indicate that the negative associations between neuroticism, self-esteem, and emotional clarity may be less pronounced among adolescents with higher levels of positive affect. Although the cross-sectional design does not allow causal inference, the results highlight the potential relevance of self-esteem and positive affect as psychological resources in adolescents with high neuroticism. These findings also underscore the importance of promoting positive emotional experiences and strengthening self-esteem as strategies for enhancing emotional well-being, with clear preventive potential for adolescents with high emotional vulnerability.

## 1 Introduction

The study of the interactions between emotional intelligence and personality variables in adolescence is crucial to understanding how adolescents manage psychological transitions at this critical stage of their development. This link, although not always evident, is highly relevant because personality traits profoundly influence how people experience and manage their emotions (Andrés et al., 2014; Augusto Landa et al., 2010; McLaughlin et al., 2011).

This article examines the role of self-esteem and positive affect as underlying mechanisms in the association between neuroticism and emotional clarity, a fundamental component of emotional intelligence. Understanding these connections may provide pathways to enhance emotional clarity in individuals with high levels of neuroticism during formative adolescence. This study addresses a gap in the literature by examining these mechanisms specifically in adolescents, a population in which this interplay has been scarcely investigated.

### 1.1 Emotional clarity in adolescence

Within Mayer and Salovey’s ability model of emotional intelligence (Mayer and Salovey, 1997; Mayer et al., 2008), emotional clarity is a specific component of the broader domain of emotional understanding, focused on the capacity to accurately identify and differentiate one’s own emotional states. It does not encompass more complex processes such as emotional blends, transitions, or the interpretation of others’ emotions, which are part of emotional understanding in its full sense. Emotional clarity refers to internal experience, is intrapersonal in nature, and is expressed as a subjective perception (“I know what I feel,” “I understand my emotions”).

To assess this construct, Salovey and Mayer developed the Trait Meta-Mood Scale (Salovey et al., 1995), a self-report questionnaire that evaluates individuals’ perceptions of their own emotional abilities: emotional attention, emotional clarity, and emotional repair. These components are interconnected (Salovey et al., 1995): effective emotional regulation requires adequate emotional understanding, which in turn depends on accurate emotional perception.

Despite the close relationship among these components (attention, clarity, and repair), the present study focuses specifically on emotional clarity, which involves investigating, categorizing, and retrospectively exploring one’s own emotions (Fernández-Berrocal et al., 2004; Martínez et al., 2011; Mayer and Salovey, 1997; Sánchez-Cabrero et al., 2022; Vanuk et al., 2019), a process that is fundamental for understanding and regulating emotions effectively (Fernández-Berrocal et al., 2004). Emotional clarity enables individuals to explain why an emotional state occurs, integrating emotion with reasoning and, consequently, making better decisions. This distinguishes it from emotional self-awareness, which simply refers to the ability to perceive one’s emotional states.

These characteristics make emotional clarity particularly relevant during adolescence, a period marked by heightened affective intensity, an identity still in formation, and an emotional regulation system that has not yet reached maturity. In this context, the ability to accurately identify and understand one’s emotional states functions as a protective factor against the emotional confusion characteristic of this stage (Mann et al., 2004). Higher emotional clarity is associated with better psychological adjustment, more effective emotional regulation, and lower vulnerability to problems such as anxiety, rumination, or depressive symptoms (Salovey et al., 1995; Calero et al., 2018). It also promotes more stable interpersonal relationships and decision-making that is more consistent with personal values (Jang and Lee, 2014; Mónaco et al., 2017), aspects that are especially relevant at a time when social belonging and identity construction play a central role. Therefore, studying emotional clarity in adolescence allows for a better understanding of the mechanisms that support healthy socioemotional development and, in this study, helps explain how variables such as neuroticism, self-esteem, and positive affect interact to shape emotional functioning during this developmental stage.

Given the particular relevance of emotional clarity during adolescence, it is essential to consider the broader social context in which today’s adolescents develop. Recent social transformations have substantially altered the emotional development of adolescents (Odgers and Jensen, 2020; Oliva, 2003; Sawyer et al., 2018; Steinberg, 2014). and, consequently, variables such as neuroticism, self-esteem, positive affect, and emotional clarity. The prolongation of adolescence and the increase in academic and social demands may intensify affective instability and foster higher levels of neuroticism. The negative social representation of adolescents in the media, together with greater exposure to social comparison in digital environments, can undermine self-esteem and reduce positive affect. Likewise, rapid cultural and technological changes, along with the growing diversity of social models, complicate identity formation and may generate greater emotional confusion, affecting the development of emotional intelligence skills, particularly emotional clarity, which requires supportive and validating environments to consolidate. Finally, changes in family structures, characterized by more democratic but also more permissive or indifferent parenting styles, directly influence emotional stability and the acquisition of socioemotional competencies. Overall, this contemporary context shapes new patterns of interaction among neuroticism, self-esteem, positive affect, and emotional clarity, underscoring the relevance of examining these variables in today’s adolescents.

### 1.2 Neuroticism and positive affect in adolescents

The Big Five model (Costa and McCrae, 1992; McCrae and Costa, 1996) identifies neuroticism as one of the main personality variables along with extroversion, responsibility, agreeableness, and openness to experience. All of these traits traditionally assessed using the Big Five Inventory (John et al., 1991, 2008) have been related to multiple aspects of emotional intelligence. For example, extroversion is associated with greater experience of positive affect and interpersonal skills, which facilitates emotional regulation and social relationships (Costa and McCrae, 1992). Similarly, openness to experience is associated with greater curiosity, cognitive flexibility, and emotional creativity, which can enhance understanding and management of emotions (DeYoung, 2010). Kindness and responsibility also play a role in emotional management, as they are related to empathy and self-discipline, two key factors for emotional management and relational well-being (Bar-On and Parker, 2000; Goleman, 1995).

Neuroticism is a trait that ranges from emotional stability to negative affectivity, including feelings of guilt, sadness, fear, or nervousness (John et al., 2008). It is therefore strongly linked to emotional instability and psychological distress, factors that are directly related to difficulties in emotional regulation and clarity. Despite the importance of the traits mentioned above, it is particularly relevant to focus on neuroticism because of its profound negative impact on emotional well-being. Moreover, this specific focus allows investigating how protective factors, such as positive affect and self-esteem, can mitigate its negative effects on emotional clarity, a central component of emotional intelligence. Understanding this interaction is crucial, given that adolescents with high levels of neuroticism are particularly vulnerable to experiencing emotional instability, and the promotion of protective factors could be key to their positive emotional development (Augusto-Landa et al., 2011).

It is interesting to note that one of the underlying emotional factors that has been shown to impact the negative aspects of neuroticism is positive affect. Positive affect refers to the degree to which a person experiences pleasant emotions, feelings, and sensations, such as joy, happiness, interest, or alertness. In terms of anxiety and depression, some researchers suggest that, while a high degree of negative affect along with a low level of positive affect is linked to anxiety, having minimal amounts of positive affect itself is associated with depression (Sánchez and Molero, 2014). In this study, positive affect is considered a state variable that fluctuates according to context and specific times (Watson et al., 1988) and, as such, is associated with the performance of various activities. This definition is appropriate in the context of adolescence, a stage characterized by intense emotional changes, where positive affect can vary significantly depending on the circumstances. However, in other research, positive affect has also been found to be a relatively stable personality trait (McCrae and Costa, 2008). People with lower levels of neuroticism tend to experience positive affect more frequently, suggesting a possible interaction between these two dimensions over time (Joshanloo, 2023; Soto, 2015; Steel et al., 2008).

In this sense, it would be valuable to investigate the role of positive affect in the connection between neuroticism and emotional clarity, particularly during adolescence, when emotional intensity, the acquisition of emotional regulation skills and the development of social identity are prominent.

### 1.3 Neuroticism, self-esteem, and emotional clarity in adolescents

Another fundamental aspect of personality that plays a key role in adolescence is self-esteem. Self-esteem can be characterized as a person’s evaluation of himself or herself, an evaluation that will condition his or her attitudes (Rosenberg, 1965). Given its relevance, it is essential to examine whether self-esteem positively influences the link between neuroticism and emotional clarity, facilitating the resolution of complex problems and the understanding of one’s emotional states. Available research indicates that those with significant levels of neuroticism are also more likely to have low self-esteem scores (Bleckmann et al., 2012; Judge et al., 2002; Li et al., 2022; Liu, 2012; Oropesa-Ruiz et al., 2022; Vazsonyi et al., 2015; Wang and Kong, 2014; Zeigler-Hill and Vonk, 2015) and, therefore, are more exposed to situational influences (Anderson et al., 2012), being more sensitive to negative social evaluations (Edler et al., 2022; Evans et al., 2016). This emotional vulnerability can hinder the development of effective emotional regulation strategies and the strengthening of emotional intelligence, which is especially relevant during adolescence, a period of intense socialization and identity formation (Mann et al., 2004).

Based on this evidence, this article addresses two main objectives: to analyze the role of self-esteem in the relationship between neuroticism and emotional clarity, and to examine the association between positive affect and lower levels of neuroticism together with higher self-esteem. The selection of these variables is grounded in a theoretical framework indicating that neuroticism is a vulnerability trait that tends to erode self-esteem (Arens and Preckel, 2025; Costa and McCrae, 1992; Sowislo and Orth, 2013), which in turn directly influences adolescents’ ability to identify and understand their own emotional states (Ciarrochi et al., 2007). For this reason, self-esteem is examined as a mediating variable, as it represents the psychological mechanism through which neuroticism may affect emotional clarity. Complementarily, positive affect is considered a moderating variable, as it functions as a protective factor capable of buffering the impact of neuroticism on emotional adjustment (Tugade and Fredrickson, 2004). Moreover, positive affect contributes to strengthening self-esteem by fostering more benign self-interpretations, promoting experiences of self-efficacy, and facilitating supportive social relationships, in line with the Broaden-and-Build Theory (Fredrickson, 2001; Lyubomirsky et al., 2005). Recent studies continue to support these associations in both adolescent (Oropesa-Ruiz et al., 2022) and adult samples (Yao, 2020), although no research to date has examined neuroticism, self-esteem, positive affect, and emotional clarity jointly in adolescent populations.

Regarding the connection between neuroticism and emotional clarity, studies with large adolescent samples have shown that emotional clarity was negatively correlated with neuroticism (Salguero et al., 2010; Thompson et al., 2015), indicating that individuals with high levels of neuroticism tend to have more difficulties in understanding and regulating their emotions. In addition, emotional clarity functioned as a protective variable in situations of depression risk. In this line, in the research of Augusto-Landa et al. (2011) with a sample of university women, who presented negative inferential styles, emotional clarity buffered the impact of stress on depressive symptoms. Similarly, when analyzing suicide risk and its relationship with emotional clarity in the adult population, other authors found that high scores in emotional clarity correlated with low scores in suicidal risk (Gómez-Romero et al., 2018; Tabares et al., 2020; Wang et al., 2016). In turn, Yildirim et al. (2022) recently suggested that emotional clarity attenuates the connection between anxiety and depression in adolescence.

As can be seen, research supports the relationship between neuroticism and emotional clarity from the perspective of negative affect. Thompson et al. (2015) clarified that when neuroticism was associated with major depressive disorder, high scores on neuroticism were only associated with a lower understanding of negative emotions, but not with positive emotions. An additional factor in the relationship between neuroticism and emotional clarity is positive affect, defined as the tendency to experience pleasant emotions, such as joy and enthusiasm (Watson et al., 1988). In this sense, the experience of positive emotions could protect against the negative effects of neuroticism and promote healthy emotional development. Indeed, positive affect have been significantly related to well-being (Lucas et al., 2003; Pinedo et al., 2017), resilience (Fredrickson et al., 2003), self-esteem, and perceived treatment by others (Dizén and Berenbaum, 2011; Gómez-Baya et al., 2019), while acting as a buffer against psychological stress (Gloria and Steinhardt, 2014).

On the other hand, previous research has shown a positive relationship between self-esteem and emotional clarity (Fernández-Ozcorta et al., 2015; Guasp-Coll et al., 2020; Valiente-Barroso et al., 2020) suggesting that people with high self-esteem have a better understanding of their emotions and are better able to handle stress and external influences (Brackett and Mayer, 2003). This association has been observed consistently across different populations and relates to broader indicators of psychological well-being. However, the relationship between these two constructs is not always linear. Guil et al. (2019) found that, in college students with high levels of trait anxiety, high self-esteem could be associated with lower emotional clarity, making self-esteem a risk factor that hinders emotional introspection. That is, in some cases, high self-esteem may hinder emotional introspection, as individuals may avoid exploring negative emotions to maintain a positive self-image. This finding suggests that self-esteem, when combined with factors such as anxiety or neuroticism, may act in complex and even contradictory ways in terms of emotional clarity (Guil et al., 2019).

Despite these complex interactions, the literature has shown that self-esteem in most cases is a protective factor, facilitating the development of emotional and social skills and contributing to greater emotional resilience (Fredrickson et al., 2003). This is consistent with previous studies showing a significant and positive relationship between self-esteem and emotional clarity among adolescents (Rey et al., 2011; Ruvalcaba-Romero et al., 2017). Gloria and Steinhardt (2014) positive affect further supports emotional adjustment in adolescents. This is especially relevant during adolescence, a stage marked by rapid development of identity and emotional regulation skills (Steel et al., 2008). At this stage, higher self-esteem and positive affect can buffer the negative effects of neuroticism, facilitating better emotional clarity and thus a greater ability to manage emotions.

From this perspective, we can expect, first, that an adolescent’s level of neuroticism will interfere with his or her emotional clarity, making it more difficult to understand and organize emotional experiences (Watson et al., 1988; Salguero et al., 2010; Salovey et al., 1995). Emotional clarity is also associated with more stable positive affective states. In addition, self-esteem is expected to mediate the relationship between neuroticism and emotional clarity, as lower self-esteem constitutes one of the mechanisms through which neuroticism undermines emotional functioning (Rosenberg, 1965). Finally, positive affect is expected to moderate the negative effects of neuroticism, buffering its impact and promoting greater emotional stability (Fredrickson et al., 2003; Gloria and Steinhardt, 2014). Based on previous research, these assumptions are integrated into a model that seeks to validate the link between neuroticism and emotional clarity during adolescence.

### 1.4 The present study

Based on the theoretical framework described above, the present study aims to examine the joint contribution of neuroticism, self-esteem, positive affect, and emotional clarity during adolescence.

The present study has the following objectives:

1. To examine whether self-esteem mediates the relationship between neuroticism and emotional clarity.
2. To analyze whether positive affect moderates the relationship between neuroticism and emotional clarity.
3. To examine whether positive affect moderates the relationship between neuroticism and self-esteem.
4. To integrate these effects into a comprehensive model linking neuroticism, self-esteem, positive affect, and emotional clarity in adolescence.

Based on these objectives, the following hypotheses are proposed:

*Hypothesis 1*. Self-esteem will mediate the relationship between neuroticism and emotional clarity, such that higher neuroticism will be associated with lower self-esteem, which in turn will predict lower emotional clarity.

*Hypothesis 2*. Positive affect will moderate the relationship between neuroticism and emotional clarity. Specifically, the negative association between neuroticism and emotional clarity will be weaker at higher levels of positive affect.

*Hypothesis 3*. Positive affect will moderate the relationship between neuroticism and self-esteem, buffering the negative impact of neuroticism on adolescents’ self-evaluations.

*Hypothesis 4*. Higher levels of positive affect will be associated with higher self-esteem, and this association will be related to greater emotional clarity.

## 2 Method

### 2.1 Participants

The study encompassed a sample of 742 Spanish adolescents, 45.1% of whom were male and 51.5% female, with ages ranging from 13 to 19 years (*M* = 15.63, SD = 1.24). All participants were high school students. The required sample size was determined using GPower 3.1. Following Cohen’s (1988) guidelines for effect size conventions, we specified a small-to-medium effect size (*f*^2^ = 0.05), a statistical power of 0.80, and a significance level of *α* = 0.05 for the planned regression, mediation, and moderated mediation analyses. Assuming four predictors in the most complex regression model, GPower indicated a minimum required sample of approximately 244 participants. The final sample of 742 adolescents therefore exceeded the recommended size, ensuring adequate statistical power for all analyses.

### 2.2 Measures

#### 2.2.1 Neuroticism

The Neuroticism subscale of the Big Five Inventory (BFI; John et al., 1991, 2008) was implemented. It consists of eight items, rated on a Likert-type scale ranging from 1 (strongly disagree) to 5 (strongly agree). Neuroticism spans from a stable emotional state to negative emotions such as anxiety, nervousness, sadness, or tension (for example: “Is relaxed, handles stress well”, “Worries a lot”, “Can be moody”). The scale demonstrated satisfactory psychometric properties (Rey et al., 2011; Rosenberg, 1965). In this study, the alpha value for the Neuroticism scale was also satisfactory (*α* = 0.75).

#### 2.2.2 Emotional clarity

We used the emotional clarity subscale of the Trait Meta-Mood Scale (TMMS-24; Fernández-Berrocal et al., 2004), modified from the Trait Meta-Mood Scale (TMMS; Salovey et al., 1995), in which emotional intelligence is considered a set of skills that can be developed and educated. The emotional clarity subscale measures an individual’s perception of their understanding of their own emotional states (for example: “I almost always know how I feel”, “I usually know my feelings about people”, “I often realize my feelings in different situations”), using eight items rated on a Likert scale ranging from 1 (strongly disagree) to 5 (strongly agree). The scale demonstrated satisfactory psychometric properties in the adolescent population (Ruvalcaba-Romero et al., 2017). In this study, Cronbach’s alpha was good (*α* = 0.88).

#### 2.2.3 Self-esteem

The Rosenberg Self-esteem Scale (Rosenberg, 1965) was implemented in the present study. This scale comprises ten items used to compute an overall self-esteem score based on the individual’s assessment of their personal characteristics, with statements such as “I am convinced that I have good qualities”, “I have a positive attitude towards myself”, “In general, I am satisfied with myself”. Adolescents respond on a Likert-type scale ranging from 1 (strongly disagree) to 4 (strongly agree). Global self-esteem, according to this approach, encompasses feelings of self-worth and self-respect. This scale demonstrated satisfactory psychometric properties with adolescent samples (Gnambs et al., 2018). In our study, the internal consistency of the scale was good (*α* = 0.87).

#### 2.2.4 Positive affect

The Positive Affect Schedule from the Positive and Negative Affect Schedule (PANAS; Watson et al., 1988) was used in this study. Positive Affect is measured through 10 items rated on a Likert scale ranging from 1 (very little or not at all) to 5 (extremely) (for example: “Interested”, “Excited”, “Strong”). High scores indicate a greater presence of positive emotions. Various studies demonstrated its good psychometric properties (Carvalho et al., 2013; Seib-Pfeifer et al., 2017). In the present study, a score of *α* = 0.76 was obtained.

### 2.3 Procedure

A cross-sectional study design was utilized for data collection. The directors of several Secondary Education centers participating in the study were contacted to explain the study’s objectives, methodology, and data usage, thereby obtaining their informed consent. The study was reviewed and approved by the institutional research ethics committee.

The research team arranged visits to the schools to collect data via questionnaires. The assessments were administered in the students’ regular classrooms, with their class teacher present. The students were informed that their participation was entirely voluntary and were provided with all necessary instructions. They were also given additional information about the confidentiality and anonymity of the data-handling process. Following the ethical standards of the Helsinki Declaration, informed consent was obtained from both parents or guardians and participants.

### 2.4 Data analytic strategy

Version 28 of the Statistical Package for the Social Sciences (SPSS) and version 4.1 of the PROCESS macro (Hayes and Montoya, 2017; Hayes, 2022) were used to examine the data. Firstly, Pearson’s correlation coefficient was calculated to conduct bivariate descriptive and correlational analysis between the study variables. Pearson’s correlation coefficient was interpreted as follows: *r* = 0.10 indicated a small effect size, *r* = 0.30 indicated a medium effect size, and *r* = 0.50 or greater indicated a large effect size (Cohen, 1992). Next, a moderated mediation model was assessed for the connection between Neuroticism and Emotional Clarity, with Positive Affect serving as a moderating variable and Self-esteem as a mediating variable, using PROCESS Model 8 (Igartua and Hayes, 2021). Confidence intervals were calculated based on 10,000 samples.

## 3 Results

### 3.1 Descriptive and correlational analysis

The means, standard deviations, and Pearson correlation coefficients for the variables analyzed are shown in Table 1. Results indicated that neuroticism was adversely associated with emotional clarity, self-esteem, and positive affect. In contrast, emotional clarity showed a positive correlation with self-esteem and positive affect. Finally, self-esteem correlated significantly and positively with positive affect.

**Table 1:** Bivariate correlations between neuroticism, emotional clarity, positive affect, and self-esteem.

	*M*	SD	Skewness	Kurtosis	Shapiro–Wilk (*p*)	1	2	3	4
1. Neuroticism	2.96	0.75	0.16	−0.37	0.12				
2. Emotional clarity	24.40	7.32	0.11	−0.67		−0.308**			
3. Self-esteem	30.49	6.42	−0.65	0.15		−0.343**	0.318**		
4. Affection positive	23.57	3.65	0.60	6.73		−0.223***	0.297**	0.454**	

*N* = 403 (listwise). The total sample consisted of 742 participants; however, 339 cases presented missing data in one or more variables and were excluded from the descriptive and correlational analyses. ^**^p < 0.01; ^***^p < 0.001; M, mean; SD, standard deviation.

In addition to these correlational analyses, skewness and kurtosis values were computed to examine the distributional properties of the study variables. Emotional Clarity and Neuroticism showed skewness and kurtosis values close to zero, indicating approximately symmetric distributions. Self-Esteem displayed a slight negative skew, whereas Positive Affect exhibited a markedly elevated kurtosis, suggesting a more peaked distribution with heavy tails.

Normality was assessed using the Kolmogorov–Smirnov and Shapiro–Wilk tests. All variables yielded statistically significant results (*p* < 0.05), indicating departures from the normal distribution. Given the large sample size (*N* = 403) and the sensitivity of normality tests to minor deviations, these findings were expected and are consistent with the distributional indices reported.

Despite these deviations from normality, the subsequent moderated mediation analyses were conducted using bootstrapping procedures, which do not rely on the assumption of normality and are considered robust for large samples. Zero-order correlations among the variables are presented in Table 1.

### 3.2 Moderated mediation analysis

A moderated mediation analysis was performed to evaluate the relationship between neuroticism with self-esteem included as a mediating variable and positive affect as a moderating variable. As seen in Table 2, in the first phase, neuroticism negatively predicted self-esteem, in the second phase positive affect did not predict self-esteem and in the third phase, the synergy between neuroticism and positive affect positively predicted self-esteem.

**Table 2:** Coefficients for the conditional process model.

Antecedent		Consequent				
		(*M*) Self-esteem			*Y* (Emotional clarity)	
		*β*	*t*		*β*	*t*
(*X*) Neuroticism	a_1_	−9.25***	−3.67	c_1_′	−4.66	1.52
(*M*) Self-esteem				b	0.22***	3.82
(*W*) Positive affect	a_2_	−0.03	−0.11	c_2_′	1.21**	3.17
(*X* × *W*) Neuroticism × Positive affect	a_3_	0.29**	2.82	c_3_′	−0.27*	−2.15
Constant	i_M_	38.16		i_y_	−6.22	
*R*^2^ = 0.31*** F(3, 399) = 61.51, *p* < 0.001		*R*^2^ = 0.20*** F(4, 398) = 25.35, *p* < 0.001				

^*^p < 0.05; ^**^p < 0.01; ^***^p < 0.001.

The dependent variable’s conditional influence on the independent variable was then calculated at various values of the moderator variable using the Pick-a-Point approach. Table 3’s data shows that the moderating effect of positive affect is present in both low, medium, and high values. The Johnson-Neyman technique was employed to determine the point at which the moderator variable begins to significantly influence the model. The findings indicated that positive affect started to significantly function as a moderating variable from a value of 27.00. Below this value, 86.35% of the adolescent sample was found, with the remaining 13.64% above it.

**Table 3:** Conditional effects of the predictor on the values of the moderator variable and conditional direct and indirect effects for the conditional process.

	Effect	Boot SE	CI 95%
Positive affect	Conditional effects		
Self-esteem			
20.00	−3.30	0.54	[−4.37, −2.23]
24.00	−2.11	0.38	[−2.86, −1.36]
27.00	−1.22	0.51	[−2.23, −0.20]
Emotional clarity			
20.00	−0.80	0.67	[−2.13, 0.53]
24.00	−1.89	0.47	[−2.82, −0.96]
27.00	−2.71	0.62	[−3.93, −1.49]

Indirect effect			Direct effect		
*W*	(a_1_ + a3W)b	95% bootstrap CI	c′1 + c′3 W	SE	*p*
20	−0.75	0.26	−0.80	0.67	0.2372
24	−0.48	0.18	−1.89	0.47	0.0001
27	−0.28	0.15	−2.71	0.62	0.0000

Indirect effect			Direct effect		
*W*	(a_1_ + a3W)b	95% bootstrap CI	c′1 + c′3 W	SE	*p*
20	−0.75	0.26	−0.80	0.67	0.2372
24	−0.48	0.18	−1.89	0.47	0.0001
27	−0.28	0.15	−2.71	0.62	0.0000

^***^p < 0.001.

SE, standard error; CI, 95% confidence interval.

In relation to emotional clarity, the results indicate that in the initial phase, neuroticism did not predict emotional clarity. In the second phase, self-esteem was found to positively predict emotional clarity. In the third phase, positive affect was observed to positively predict emotional clarity. Lastly, in the fourth phase, the interaction between neuroticism and positive affect was seen to negatively predict emotional clarity (see Table 2).

The conditional effect of the independent variable on the dependent variable was subsequently evaluated using the Pick-a-Point technique at various levels of the moderator variable. As shown in Table 3, the moderating effect of positive affect is apparent at both medium and high levels.

The Johnson-Neyman technique was employed to determine the point at which the moderator variable begins to significantly influence Model 1. The results showed that positive affect began to significantly serve as a moderating variable from a value of 21.21. Below this value, 28.28% of the adolescent sample was found, with the remaining 71.71% above it.

**Figure 1:**
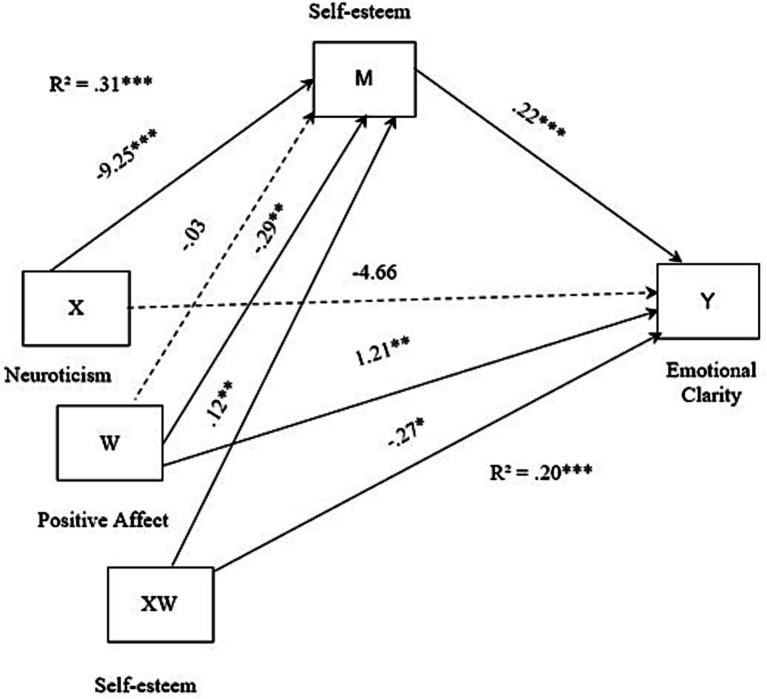
Statistical diagram of the conditional process model.

The moderate mediation index was 0.0682, SE = 0.0298, 95% CI = [0.0198, 0.1350]. This indicates that the negative effect of neuroticism on emotional clarity was mediated by self-esteem, with the effect being less significant when positive affect was medium to high (see Table 3). The final model of the analysis is shown in Figure 1.

## 4 Discussion

The results of this study offer a more integrated understanding of how neuroticism relates to emotional clarity in adolescence, highlighting the central role of self-esteem and positive affect in this process. Overall, the findings support the proposed moderated mediation model, showing that self-esteem functions as a key explanatory mechanism linking neuroticism to emotional clarity, while positive affect modulates the strength of these relationships.

First, the study confirmed that self-esteem mediates the relationship between neuroticism and emotional clarity, consistent with Hypothesis 1. Adolescents with higher levels of neuroticism tend to exhibit lower self-esteem, which in turn is associated with a reduced ability to identify and understand their emotions. This pattern suggests that self-esteem constitutes a fundamental psychological resource that can buffer the negative impact of neuroticism on emotional understanding.

Moreover, the results showed that positive affect moderates both the relationship between neuroticism and emotional clarity (Hypothesis 2) and the relationship between neuroticism and self-esteem (Hypothesis 3). In both cases, higher levels of positive affect attenuate the detrimental effects of neuroticism, acting as a protective factor. Thus, adolescents who experience positive emotions more frequently appear to maintain more stable self-esteem and greater emotional clarity, even when they exhibit high levels of neuroticism.

It is important to emphasize that fostering the development of positive affect in situations of neuroticism does not imply that adolescents cannot experience negative affect or that these emotions should be suppressed. Rather, the goal is for these negative affects to be recognized, accepted, and eventually transformed into more positive emotional experiences. This ability to recontextualize adverse emotions may be crucial for helping adolescents develop a richer and more nuanced emotional understanding of themselves. By learning to navigate their emotions, young people can strengthen their emotional resilience and improve their overall well-being.

The findings align with previous research on the intensity of positive affect and its relationship with neuroticism (Meesters et al., 2007), as well as with studies showing a connection between positive affect and emotional clarity (Mónaco et al., 2017). This highlights the protective role of positive affect in enhancing adolescents’ mental health and well-being.

There are several explanations for the fact that positive affect reduces the likelihood of low emotional clarity in individuals with neuroticism. One possible explanation is that the aspect of neuroticism that hinders the identification and understanding of one’s emotions is, to a greater extent, the experience of negative affect. Due to their aversive nature, negative emotions are more challenging to accept and sometimes lead to avoidance and lack of recognition (Smith and Ellsworth, 1985). However, positive emotions, given their pleasant and reinforcing nature, are more likely to be consciously recognized and accepted (Mónaco et al., 2017). Therefore, although individuals who generally experience emotions with greater intensity may struggle to gain perspective on their own emotions (their awareness tends to easily merge with their emotions and thoughts), the experience of positive affect may serve as a facilitating context to improve awareness and understanding of their internal world (Cohn and Fredrickson, 2010).

The results also corroborated Hypothesis 3, as positive affect and neuroticism jointly predicted self-esteem. In this regard, positive affect emerged as a protective factor, buffering the negative impact of neuroticism on adolescents’ self-evaluations. Moreover, this protective effect extended to emotional clarity, since higher levels of positive affect were associated with greater self-esteem, which in turn predicted higher emotional clarity, thereby confirming Hypothesis 4. This suggests that adolescents who frequently experience positive emotions are more likely to maintain their self-esteem even when they exhibit high levels of neuroticism, and that this emotional context facilitates a clearer understanding of their internal states. These findings are consistent with previous research linking high neuroticism to negative affect (Karaaslan et al., 2020; Shi et al., 2015), as well as with studies showing that neuroticism is associated with lower self-esteem (Judge et al., 2002; Liu, 2012). The positive association between self-esteem and emotional clarity has also been documented (Guasp-Coll et al., 2020), supporting the present results. Additionally, evidence suggests that parenting strategies based on humor or optimism can enhance adolescents’ positive affect and life satisfaction, further highlighting the relevance of positive emotional experiences for psychological well-being.

The study provides relevant empirical evidence by simultaneously integrating processes related to personality, affect, and emotional self-concept in an adolescent population, a developmental period that is particularly sensitive for socioemotional growth. The confirmation of the proposed hypotheses strengthens theoretical models that conceptualize positive affect as a key modulating factor in the relationship between personality traits and emotional competencies, expanding the understanding of the mechanisms that explain why some adolescents with high levels of neuroticism exhibit better emotional outcomes than others. Moreover, the findings highlight the central role of self-esteem as an explanatory mechanism, offering a clear pathway for the design of interventions. In applied terms, the study supports the need to promote educational programs that incorporate emotional education, the strengthening of self-esteem, and training in positive affective experiences, both in school and family contexts, with the aim of reducing the emotional vulnerability associated with neuroticism.

Building on these results, future research could examine how the interactions among neuroticism, positive affect, self-esteem, and emotional clarity evolve longitudinally throughout adolescence, analyzing whether the protective effects of positive affect and self-esteem are maintained, intensified, or weakened over time. It would also be relevant to evaluate the effectiveness of specific interventions aimed at promoting positive affect and strengthening self-esteem, assessing their impact on reducing the negative effects of neuroticism in educational and family settings. Additionally, extending the study to different cultural contexts would allow for testing the generalizability of the findings, taking into account variations in parenting styles, emotional expression, and social norms. Collectively, these future lines of inquiry will contribute to the development of more comprehensive models and more precise interventions that enhance the emotional well-being of adolescents with high levels of neuroticism.

These results align with previous work that has identified both positive affect and self-esteem as protective resources against vulnerability traits such as neuroticism (Fredrickson, 2001; Orth and Robins, 2014). The mediation of self-esteem and the moderation of positive affect observed in this study are consistent with research highlighting their role in emotional regulation and psychological adjustment during adolescence. Moreover, the present study offers a unique contribution by showing that these resources not only buffer vulnerability but also enhance adolescents’ emotional clarity, supporting a more adaptive understanding and management of their emotional experiences. Moreover, positive affect enhances resilience in contexts of vulnerability, and self-esteem helps maintain emotional stability, thereby reinforcing their protective function.

## 5 Limitations

This study presents several limitations that should be considered when interpreting the findings. First, the cross-sectional design prevents establishing causal relationships among the variables, as it only allows the identification of associations without determining the direction of effects. Additionally, although the instruments used are widely validated, the measurement of positive affect through the PANAS is sensitive to contextual and momentary mood fluctuations. This sensitivity is not a limitation of the instrument itself but reflects the nature of positive affect as a state variable, whose variability is inherent to any assessment of affective states. Furthermore, although the sample is balanced by gender, an important methodological strength, previous research has documented gender differences in average levels of neuroticism (Hou et al., 2024; Weisberg et al., 2011) and, less consistently, in emotional clarity. These differences may influence the interpretation of the results and their extrapolation to populations with different gender distributions. Future studies employing longitudinal designs and broader samples would help clarify how these relationships operate across different groups.

## 6 Conclusion

The results obtained confirm the four proposed hypotheses and indicate that neuroticism, positive affect, self-esteem, and emotional clarity form a dynamic system of mutual influences during adolescence. First, the findings show that self-esteem mediates the relationship between neuroticism and emotional clarity, such that higher levels of neuroticism are associated with lower self-esteem, which in turn predicts lower emotional clarity. Likewise, positive affect is shown to moderate both the relationship between neuroticism and emotional clarity and the relationship between neuroticism and self-esteem, attenuating the negative impact of neuroticism on both variables. Finally, higher levels of positive affect are confirmed to be associated with higher self-esteem, which contributes to greater emotional clarity. Taken together, these findings indicate that positive affect and self-esteem function as protective resources that buffer the effects of neuroticism and promote more adaptive emotional functioning.

Taken together, the results show that emotional clarity in adolescents does not depend solely on neuroticism, but also on personal resources such as self-esteem and positive affect. These findings highlight the importance of promoting positive emotional experiences and strengthening self-esteem as strategies for enhancing emotional well-being, with clear preventive potential for adolescents with high emotional vulnerability.

## Data Availability

The original contributions presented in the study are included in the article/supplementary material, further inquiries can be directed to the corresponding authors.
